# Reproductive Behavior and Fitness Components in Male *Drosophila melaogaster* are Non-Linearly Affected by the Number of Male Co-Inhabitants Early in Adult Life

**DOI:** 10.1673/031.011.6701

**Published:** 2011-05-25

**Authors:** B. Nandy, N. G. Prasad

**Affiliations:** Indian Institute of Science Education and Research Mohali, MGSIPA Complex, Sector 26, Chandigarh 160 019, India

**Keywords:** copulation duration, male fitness, mating latency, sperm defense

## Abstract

Although multiple lines of evidence suggest that early adult life is very important in shaping the reproductive behavior of males, few studies have looked at the fitness consequences of the variation in reproductive behavior induced by differences in early life experience of males. Using a long term laboratory adapted population of *Drosophila melanogaster* Meigen (Diptera: Drosophilidae), early life experience, in terms of co-inhabitant numbers, was found to affect male mating behavior and at least one fitness component. However, in contrast to previous studies, a non-linear relationship was found between early life experience and fitness components and a significant effect of co-inhabitant number on copulation duration and sperm defense. Both these traits showed a sharp increase as the co-inhabitant numbers changed from 1 to 16. However, there was a decline in the trait values as the co-inhabitant number increased further. The probable causes for the observed non-linear pattern of responses are discussed.

## Introduction

In sexually reproducing species with little or no parental care, male fitness depends largely on the number of matings and the average number of progeny sired from each mating (Bateman 1948). Mating success of males in many species is largely dependent on the ability of the males to perform a set of complex behaviors, which together are termed mating behavior. Fruit flies are one of the best model systems to study male reproductive behavior because they have a promiscuous mating system with considerable genetic variation for an elaborate male reproductive behavioral repertoire (Moehring and Mackay 2004).

Previous studies have documented the ‘plasticity’ of male mating behavior in various species of *Drosophila.* Immature *Drosophila* males that elicited courtship from mature males had significantly lower mating latency (time taken by a virgin pair to start mating) as adults compared to males that did not elicit homosexual courtship (McRobert and Tompkins 1988). *Drosophila* housed in groups had lower mating frequencies and higher mating latency compared to flies housed singly, and males preferred females housed singly to those housed in groups (Ellis and Kessler 1975). Flies housed under light-dark cycles had greater mating success than flies housed in constant darkness (Hirsch et al. 1995). *Drosophila* maintained in an enriched environment (presence of combination of complex inanimate and social stimulation during housing) during early adult life had higher mating success than those maintained in standard environments (Dukas and Moores 2003). Thus, there is a growing body of evidence that early life experience affects male mating behavior and mating success to a great degree in *Drosophila.* Some of these findings are relevant to the theories of sperm competition (Parker 1993; Engqvist and Reinhold 2005), wherein sperm from different males compete with each other with in the female genital tract (Parker 1970). Sperm competition is considered a potent driving force for the evolution of several morphological, behavioral and physiological traits (Snook 2005). Models of sperm competition recognize two different parameters — risk and intensity (Parker 1990, 1996; Engqvist and Reinhold 2005). These models predict that males should evolve (a) mechanisms to gauge levels of sperm competition and (b) prudent ejaculate investment strategies based on varying levels of these two parameters (Engqvist and Reinhold 2005, 2006). Williams et al. (2005) provide an alternative model of sperm competition, where the degree of sperm competition is coupled with sperm allocation. They show that factors, such as cost of mating, total resource availability and degree of sperm precedence (rather than degree of sperm competition per se), can drive the evolution of sperm allocation strategy.

Empirical evidence in support of the ability of males to gauge levels of sperm competition and invest accordingly comes from diverse species of insects, including crickets, butterflies and fruit flies (Gage and Barnard 1996; Wedell and Cook 1999; Friberg 2006; Bretman et al. 2009). In *Drosophila*, males can use female mating status and the number of potential competitors to gauge levels of sperm competition. Males were found to mate longer with females that are perceived as previously mated compared to females perceived as virgins (Friberg 2006). Two recent studies have shown that males held in groups during early adult life mated longer than males held singly (Bretman et al. 2009, 2010). Thus, at least in *Drosophila,* copulation duration is a potential measure of male investment in response to perceived levels of sperm competition. Although copulation duration is considered an indicator of male ejaculate investment, results from some of the recent studies indicate that the variation in copulation duration can be attributed to variation in amount of accessory factors transferred (Friberg 2006; Bretman 2009, 2010).

In the present study, we addressed the following questions: (a) is male mating behavior in *Drosophila melanogaster* Meigen (Diptera: Drosophilidae) affected by the number of male co-inhabitants experienced early in adult life in the way predicted by the theory of sperm competition? (b) do changes in mating behavior affect male competitive fitness? Male *D. melanogaster* were exposed to different numbers of male co-inhabitants very early in their adult life and then assayed their mating latency and copulation duration. The sperm defense ability (i.e. the ability to resist displacement by sperm from other males) of the males from different treatments, was quantified as a measure of the fitness consequences of the behavior.

## Materials and Methods

A large, outbred laboratory population of *D. melanogaster* called LH_st_ (Prasad et al. 2007) was used. This population was derived by introgressing an autosomal recessive *st* (scarlet-eye) allele through repeated back-crossing into a long-term laboratory-adapted population, LH (Chippindale and Rice 2001) with red-eyed phenotype. Both populations were maintained on a 14 day discrete generation cycle at 25° C and 12:12 L:D and fed a cornmeal-molasses diet and were maintained as large populations (> 2000 individuals) to avoid inbreeding effects.

For the present experiment, eggs were collected from adult flies and dispensed into 8 dram vials containing cornmeal-molasses diet at a density of 150 eggs/vial. During peak eclosion, males were collected as very young virgins (<4 hrs post eclosion) and randomly assigned to one of five different treatments that differed in the number of males (1, 8, 16, 24 or 32 males per vial) that were held together for a period of two days after eclosion. Space within the vial was adjusted to keep the space available per individual constant across the treatments. This was done by pushing the cotton plug to different depths. A space of about 3 ml per individual was allowed between food and cotton plug. During the experiment, a single male from each treatment group was paired with a single 3-day old, virgin LH_st_ female. Each pair was observed individually to determine the mating latency and copulation duration. All experimental males were successful in mating. After about an hour, by which time almost all flies had completed mating, the flies were separated using light CO_2_ anaesthesia. After half an hour, females were combined with control, red eyed (LH) males and allowed to interact for 20 – 22 hours, after which the males were discarded and females were put into individual test tubes (12 mm × 75 mm) with medium and allowed to oviposit for 18 hrs. Twelve days later, the progeny were scored on the basis of their eye color. The proportion of scarlet-eyed flies gave the sperm defense (P1: proportion of progeny sired by the first male when the female has mated with two males sequentially) value of the experimental males. The fraction of the females that did not re-mate yielded a value of fidelity.

The entire experiment was done in three separate blocks, which were run on three successive days, with 15 replicates of each treatment in each block. Block means were used as the units of analysis. For sperm defense, analyses were done on both raw and arcsine square-root transformed data. Data for each of the traits measured were analysed using a two-way mixed model analysis of variance (ANOVA) with treatment as the fixed factor crossed with randomised block. Multiple comparisons were implemented using Tukey's HSD. All these analyses were done using STATISTICA for Windows.

## Results

**Figure 1.**
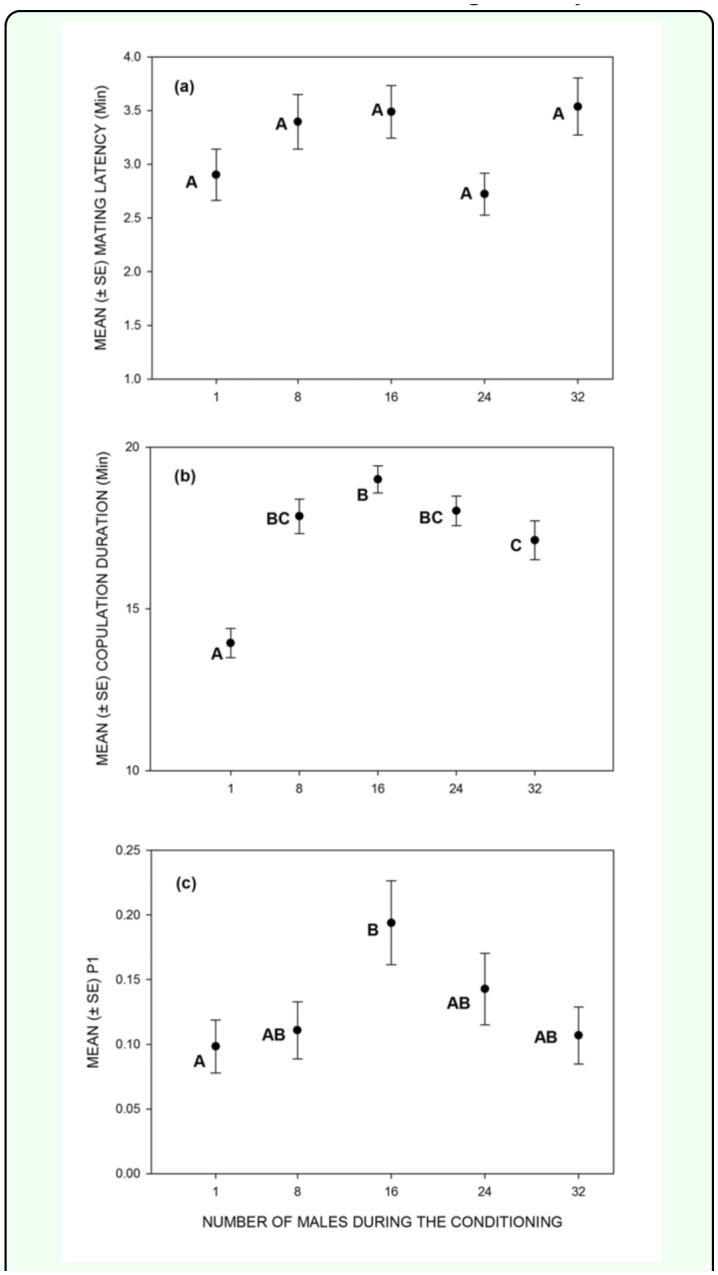
Effect of number of co-inhabitants experienced early in life on (a) mating latency, (b) copulation duration and (c) sperm defense ability (P1). Data points not sharing at least one common letter are significantly different. High quality figures are available online.

The results of the ANOVAs are summarised in Table 1. There was no significant effect of treatment on mating latency (Figure 1a). Copulation duration varied significantly across the treatments (Figure 1b). Copulation duration increased until the 16-male treatment and then decreased until the 32-male treatment. Multiple comparisons indicated that the single male treatment was significantly different from all the other treatments. Additionally, the 16-male treatment was significantly different from the 32-male treatment.

Treatment had a significant effect on sperm defense (Table 1). The P1 values showed a distribution similar to that of the copulation duration (Figure 1c). P1 increased from single to 16-male treatment and thereafter declined until the 32-male treatment. Multiple comparisons indicated that the single male treatment was significantly different from the 16-male treatment. A linear regression of mean P1 values on mean copulation duration yielded a significant positive slope (slope = 0.015, *r*^2^ = 0.27, *p* = 0.049). No significant effect of the treatment on mating fidelity was observed.

**Table 1.**
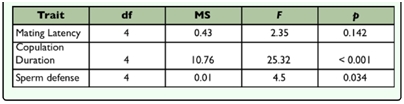
Spearman rank correlation between environmental factor (canopy density and thickness of the substrate), study area and specimens number.

## Discussion

In our study, males were exposed to increasing numbers of co-inhabitants during early adult life and then components of their reproductive behavior and fitness were measured. The results show that copulation duration, an important component of the reproductive behavior, is plastic, with the males confined with either high or low numbers of co-inhabitants showing lower copulation duration compared to males confined with intermediate numbers of coinhabitants. Moreover, the variation in copulation duration was positively correlated with an important component of male fitness, namely sperm defense ability. Males from the 16-male treatment had higher P1 values compared to either males from the single or 32-male treatment. This fitness difference among the males is not attributable to the differences in their ability to inhibit further mating. Thus, the results indicate that the number of early life co-inhabitants faced by males may affect their later life fitness by altering components of reproductive behavior.

There are several potential explanations for the observed change in copulation duration with the number of co-inhabitants. Male density prior to assay can have major effects on male courtship. *Drosophila* males held at high density tend to have lesser courtship intensity compared to males held isolated (Noor 1997). However, such density effects were ruled out in our experiment. By varying the total available volume within the containers used in the experiment the males had the same per capita space across treatments. Density effects being mediated through competition for food were also ruled out. This was because (a) the food provided in the vial was enough to support a large number of flies and (b) males do not feed much compared to the females (Stewart et al. 2005).

Increasing the number of co-inhabitants increases the chances of interactions, which has the potential to affect male reproductive behavior, quite independent of space- and food-limitation related effects. In *Drosophila,* the reproductive behavior of a male can be affected by its interactions with other sexually mature males during its early, immature stages (Gailey et al. 1982; McRobert and Tompkins 1988). However, in our experiment, all individuals were of the same age, consequently, the differences in the reproductive behavior of males from various treatments cannot be attributed to the interaction between mature and immature males. Additionally, while increased male-male interaction has considerable fitness cost (Gaskin et al. 2002), it is unlikely to be a major factor in our experiment, as males of the population used commonly show very little male-male courtship. But lack of direct observation during the experiment prevents us from confirming this prediction.

Alternatively, the observed responses may reflect the adaptive, plastic ejaculate investments by males based on their perception of the level of sperm competition. In promiscuous species, like *Drosophila,* females may mate multiply and store sperm from multiple males leading to sperm competition (Snook 2005). Increasing the number of co-inhabitants might alter the perception of sperm competition intensity or sperm competition risk in males. Theory predicts that male investment should vary based on both sperm competition intensity and sperm competition risk (Engqvist and Reinhold 2005). Such theories also distinguish between “average” (long- term average in a population) and “immediate” (in a given round of mating) levels of intensity and risk. Male investment is predicted to increase with increasing average levels of both intensity and risk. However, risk models predict increasing investment with increased immediate levels of risk, whereas intensity models predict decreasing investment with increased immediate levels of intensity (Parker et al. 1997; Engqvist and Reinhold 2005). In a recent study, Bretman et al. (2009) altered both average and immediate levels of sperm competition by varying the number of competitors that males are housed with prior to and during the assay and found that males adaptively varied their investment. Male investment increased with average levels of sperm competition but decreased with increasing immediate levels of sperm competition, an observation largely consistent with the predictions of intensity models of sperm competition. In our experiments, males were confined with cohabitants for two days and then assayed in the absence of a competitor. Hence, average sperm competition intensity/risk levels were varied but immediate sperm competition intensity/risk levels were constant and zero.

Sperm competition theory (Engqvist and Reinhold 2006) predicts increased investment with increased average levels of sperm competition intensity/risk. Our results agree partly with the predictions of sperm competition theory and the results of Bretman et al. (2009) in that the copulation duration increased as the male number increased from one to 16. The observed decline in copulation duration as male numbers increased from 16 to 32 is not in agreement with the predictions of sperm competition theory. While at present we do not have a mechanism to explain the observed decline in copulation duration at higher male numbers, there are several possibilities: (a) Group sizes in our study are larger than those of Bretman et al. (2009, 2010). The sizes of the largest groups in the study of Bretman et al. were 4 (Bretman et al. 2009) and 16 (Bretman et al. 2010), whereas it was 32 in our study. While theories suggest increased investment with increased average levels of sperm competition, it is very likely that there exists a certain limit beyond which it might not be biologically feasible for an organism to invest in larger ejaculates and/or the costs of investing in such ejaculates might be very high. In fact, alternative treatments of sperm competition (Williams et al. 2005) suggest that the ‘evolutionarily stable strategy’ level of sperm allocation decreases with increasing mating cost and strong last male precedence. However, we are not aware of any efforts to extend these alternative treatments of sperm competition to plastic ejaculate investments by the males based on perceived levels of sperm competition, (b) It is quite possible that housing males with other males for a period of time might alter their perception of both average and immediate intensity/risk, thereby making comparisons with predictions from sperm competition theory more difficult. (c) It is important to note that the theoretical predictions of Engqvist and Reinhold (2006) assume numerical competition between sperm and concern investment of sperm in different matings by the males. The ejaculate (sperm along with the seminal proteins) investment pattern might be much more complicated than what is predicted. For example, in species like *D. melanogaster*, with high last male sperm precedence and moderate level of remating frequency, theory predicts very little change in sperm investment with changing risk of sperm competition when mating with virgin females (Engqvist and Reinhold 2006). However, with increasing risk of sperm competition, males might still be selected for injecting more of the accessory gland proteins even to virgin females which might give them higher ability to defend against possible sperm displacement, which would mean an increase in the copulation duration. Hence, given that variation in copulation duration in *Drosophila* is likely to represent a variation in accessory gland proteins rather than a variation in sperm numbers and that sperm competition is affected by accessory gland proteins, the theoretical predictions of the pattern of variation in copulation duration with changing levels of sperm competition are not clear.

In conclusion, our study clearly shows that (a) reproductive behavior in male *D. melanogaster* can be non-linearly affected by the number of male co-inhabitants experienced early in adult life, and these changes in behavior are partly consistent with the predictions from theories of sperm competition and (b) these changes in behavior directly affect at least one component of male fitness, sperm defence ability.

## Abbreviations

**PI**,: sperm defence score;
**LH**,: red eye strain;
**LHst**,: scarlet eye strain
